# Examination of gammarid transcriptomes reveals a widespread occurrence of key metabolic genes from epibiont bdelloid rotifers in freshwater species

**DOI:** 10.1098/rsob.230196

**Published:** 2023-10-25

**Authors:** Alberto Ribes-Navarro, Naoki Kabeya, L. Filipe C. Castro, André Gomes-dos-Santos, Miguel M. Fonseca, Hilke Alberts-Hubatsch, Francisco Hontoria, Juan C. Navarro, Óscar Monroig

**Affiliations:** ^1^ Instituto de Acuicultura Torre de la Sal (IATS), CSIC, 12595 Ribera de Cabanes, Castellón, Spain; ^2^ Department of Marine Biosciences, Tokyo University of Marine Science and Technology, Konan 4-5-7, Minato, Tokyo, Japan; ^3^ CIMAR/CIIMAR—Interdisciplinary Centre of Marine and Environmental Research, University of Porto, Avenida General Norton de Matos, S/N, 4450-208 Matosinhos, Portugal; ^4^ Department of Biology, Faculty of Sciences, University of Porto (U. Porto), Rua do Campo Alegre S/N, 4169-007 Porto, Portugal; ^5^ Alfred Wegener Institute, Helmholtz Centre for Polar and Marine Research, Bremerhaven, Germany

**Keywords:** bdelloid rotifers, gammarids, horizontal gene transfer, long-chain polyunsaturated fatty acid biosynthesis, transcriptome

## Abstract

Previous data revealed the unexpected presence of genes encoding for long-chain polyunsaturated fatty acid (LC-PUFA) biosynthetic enzymes in transcriptomes from freshwater gammarids but not in marine species, even though closely related species were compared. This study aimed to clarify the origin and occurrence of selected LC-PUFA biosynthesis gene markers across all published gammarid transcriptomes. Through systematic searches, we confirmed the widespread occurrence of sequences from seven elongases and desaturases involved in LC-PUFA biosynthesis, in transcriptomes from freshwater gammarids but not marine species, and clarified that such occurrence is independent from the gammarid species and geographical origin. The phylogenetic analysis established that the retrieved elongase and desaturase sequences were closely related to bdelloid rotifers, confirming that multiple transcriptomes from freshwater gammarids contain contaminating rotifers' genetic material. Using the *Adineta steineri* genome, we investigated the genomic location and exon–intron organization of the elongase and desaturase genes, establishing they are all genome-anchored and, importantly, identifying instances of horizontal gene transfer. Finally, we provide compelling evidence demonstrating Bdelloidea desaturases and elongases enable these organisms to perform all the reactions for de novo biosynthesis of PUFA and, from them, LC-PUFA, an advantageous trait when considering the low abundance of these essential nutrients in freshwater environments.

## Graphical abstract

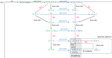

## 1. Introduction

Long-chain (≥C_20_) polyunsaturated fatty acids (LC-PUFA), and more specifically, the omega-3 (*ω*3 or *n-*3) LC-PUFA eicosapentaenoic acid (EPA, 20 : 5*n-*3) and docosahexaenoic acid (DHA, 22 : 6*n-*3), are of high relevance for animal health and development [1–5]. Aquati

c ecosystems, and particularly marine habitats, are main sources of *n-*3 LC-PUFA due to the abundance of low trophic organisms with the ability for their endogenous production (biosynthesis) [6]. Microorganisms such as photosynthetic microalgae, heterotrophic protists and bacteria have been historically regarded as primary producers of *n-*3 LC-PUFA in the ocean [7–9]. However, more recently, certain invertebrates have been also shown to contribute to *n-*3 LC-PUFA production in both aquatic and terrestrial ecosystems [10–13]. Flux of *n-*3 LC-PUFA from primary producers to upper trophic levels is crucial for marine ecosystems since fish, as all vertebrates, depend upon provision of pre-formed health beneficial *n-*3 LC-PUFA in their diet [6]. Interest in understanding the mechanisms by which living organisms biosynthesize *n-*3 LC-PUFA has been prompted in recent years due, among other reasons, to the decrease in global availability of these essential nutrients predicted to occur because of climate change [6,14,15].

**Figure 1.**
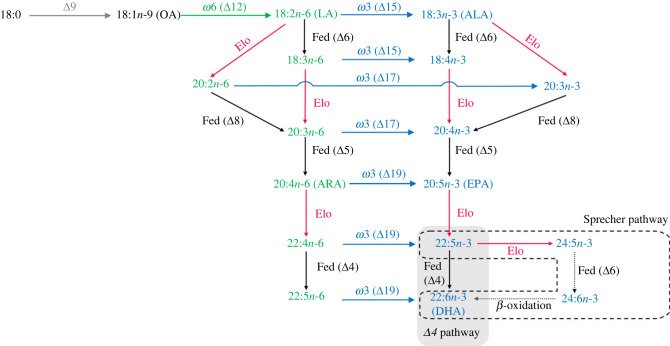
General overview of the LC-PUFA biosynthesis pathways in animals. Reactions catalysed by front-end desaturases are marked as ‘Fed’ along with their specific Δ*y* activity (*y* indicates position of double bond from the –COOH terminus of the fatty acid). Reactions catalysed by methyl-end desaturases are denoted as ‘*ωx*’ (*x* indicates position of double bond from the –CH_3_ terminus of the fatty acid), and their specific Δ*y* activity. Reactions marked with ‘Elo’ are catalysed by elongation of very long-chain fatty acid (Elovl) proteins. Two distinct pathways enabling DHA biosynthesis are possible, namely the ‘Δ4 pathway’ (grey background) and the ‘Sprecher pathway’ (dashed line).

Microorganisms can biosynthesize *n-*3 LC-PUFA using either anaerobic or aerobic metabolic pathways, each of them involving different enzymatic components [16]. The anaerobic pathways occur mostly in prokaryotes and some eukaryotic organisms, and are mainly driven by the polyketide synthase (PKS) complex [17]. However, most eukaryotic organisms including animals operate aerobic pathways enabling LC-PUFA biosynthesis through the orchestrated action of three key enzymes, namely methyl-end desaturases (*ω*x), front-end desaturases (Fed), and elongation of very long-chain fatty acid (Elovl) proteins [10,11]. Briefly, the monoene oleic acid (OA, 18 : 1*n-*9), biosynthesized from stearic acid (18 : 0) by a Δ9 desaturase occurring in virtually all living organisms [18], can be converted into linoleic acid (LA, 18 : 2*n-*6) and α-linolenic acid (ALA, 18 : 3*n-*3), which represent the simplest cases of *n-*6 and *n-*3 polyunsaturated fatty acids (PUFA), respectively (figure 1). With some exceptions [19–22], the de novo biosynthesis of *n-*6 and *n-*3 PUFA (i.e. LA and ALA) is carried out by *ω*x enzymes (figure 1). The *ω*x responsible for the biosynthesis of LA from OA are commonly known as Δ12 or *ω*6 desaturases, whereas those enabling the conversion of LA into ALA are known as Δ15 or *ω*3 desaturases (figure 1). Interestingly, *ω*3 desaturases typically recognize a variety of *n-*6 PUFA as substrates, converting them into *n-*3 products (figure 1). LA and ALA have no major biological functions themselves but serve as precursors of the physiologically important LC-PUFA by the action of further enzymes including Fed and Elovl (figure 1). Like *ω*x, Fed introduce double bonds (unsaturations) into the pre-existing fatty acyl chains, but such insertion takes place in the ‘front end’ of the fatty acid acting as substrate, more specifically between an existing double bond and the carboxyl terminus (–COOH) [23,24]. Typical activities reported in Fed include Δ4, Δ5, Δ6 and Δ8 (figure 1) [10]. Besides, Elovl catalyse the condensation reaction in the fatty acid elongation pathway that results in the extension of an existing fatty acid in two carbons [10,23] (figure 1). Both invertebrates and vertebrates possess Fed and Elovl involved in the biosynthesis of LC-PUFA from C_18_ PUFA precursors (figure 1). However, a distinctive trait among animals' LC-PUFA biosynthetic capacity is that invertebrates, but not vertebrates, can have *ω*x enabling in them the de novo biosynthesis of PUFA (i.e. LA and ALA) described above (figure 1).

Evidence supporting the capacity of invertebrates for LC-PUFA biosynthesis had been mostly collected from compositional studies and biochemical assays aiming to determine *in vivo* bioconversions of labelled fatty acid substrates [25–31]. However, the ever-increasing availability of genomic data from multiple invertebrates now offers the opportunity to identify the specific genes encoding LC-PUFA biosynthetic enzymes, thus often enabling one to distinguish the invertebrate's enzymatic capacity from that of (micro)organisms that may inhabit their hosts [32]. Studies aiming to characterize LC-PUFA biosynthetic genes from invertebrates have helped to predict the gene complement and function in certain groups [10,12,33]. Crustaceans have been the focus of particular attention due to their central roles in trophic ecology of essential fatty acids in aquatic ecosystems, as well as their commercial interest in aquaculture [34–39]. Current evidence suggests that both *ω*x and Fed enzymes occur in certain copepods [12,37,40], but are absent in other crustaceans [10]. Comparatively to desaturases, the repertoire of Elovl with functions along the LC-PUFA biosynthetic pathways in crustaceans is far more varied, with occurrence of multiple *elovl*-like genes, not only in copepods [37,40,41] but also in decapods [34,36,42,43], branchiopods [39] and gammarids [44]. Such an Elovl diversity has been hypothesized to compensate for the apparent absence of Elovl2/5 in crustaceans, a pivotal PUFA Elovl reported in molluscs and amphioxus [45–47]. Surprisingly, Ribes-Navarro *et al*. found putative *elovl2/5* sequences in freshwater gammarids, but not in marine species, an unexpected finding considering they are very closely related species, often from the same genus (e.g. *Gammarus*) [44].

The present study aimed to clarify the origin and occurrence of sequences of LC-PUFA biosynthesizing genes, such as *elovl*2/5 and further biosynthetic gene components including Fed and *ω*x in freshwater gammarids. To do so, we conducted a systematic search of diagnostic motifs characteristic of each three LC-PUFA biosynthetic enzyme families within publicly available transcriptomes from both freshwater and marine gammarids [48–55]. Our results confirmed that sequences encoding putative Elovl2/5, Fed and *ω*x are widespread in transcriptomic databases from freshwater gammarids but absent in those from marine species. We further analysed the phylogenetic relationship of the retrieved desaturases and elongases. Strikingly, we unequivocally established that they were closely related to bdelloid rotifers. Using the genome of the freshwater bdelloid rotifer *Adineta steineri* as a proxy-model, we investigated the genomic location and exon–intron organization of all the desaturase and elongase genes used as diagnostic markers. We establish they are all genome-anchored and clarify some instances of horizontal gene transfer (HGT). Finally, we provide compelling evidence that the Bdelloidea desaturase and elongase sequences encode enzymes enabling all the reaction for de novo biosynthesis of PUFA and, from them, of LC-PUFA.

## 2. Material and methods

### 2.1. Retrieval of LC-PUFA biosynthesis genes in gammarid transcriptomes

Sequences from functionally characterized LC-PUFA biosynthetic desaturases and elongases from aquatic invertebrates including annelids, molluscs and arthropods were used as queries for BLAST searches (*tblastn*) within transcriptomes from a variety of gammarids, including freshwater and marine species [48–55]. Hits were only selected for further analysis when they contained the full-length open reading frame (ORF) and their predicted protein sequences (https://www.ncbi.nlm.nih.gov/orffinder) contained specific features according to Hashimoto *et al*. [56]. Briefly, Fed had to contain three diagnostic histidine boxes (H-box) ‘HXXXH’, ‘HXXXHH’ (or ‘HXXHH’) and ‘QXXHH’, and a heme binding motif (HPGG) in the cytochrome *b*_5_ domain. Putative desaturase sequences with the third H-box ‘HXXHH’ instead of ‘QXXHH’ were not regarded as Fed based on evidence collected from other crustaceans suggesting these enzymes lack fatty acyl desaturation capacity [10,11]. Selection of *ω*x was made based on the presence of the three H-boxes as ‘HXXXH’, ‘HXXHH’ and ‘HXXHH’, and absence of cytochrome *b*_5_ domain [56]. As for elongases, the deduced amino acid (aa) sequences had to contain the conserved domains ‘KXXEXXDT’, ‘NXXXHXXMYXYY’ and ‘TXXQXXQ’, and a H-box as ‘HXXHH’.

### 2.2. Phylogeny of LC-PUFA biosynthesis genes retrieved from freshwater gammarid transcriptomes

The deduced aa sequences of the *elovl2/5*, *fed* and *ωx* retrieved from freshwater gammarid transcriptomes were used for phylogenetic analysis, along with a wide range of previously characterized genes from vertebrates and invertebrates [12,37,44]. Putative desaturase and elongase sequences from Rotifera transcriptomes (PRJNA295486-295489) were also included in the analysis [57]. Besides, for the Fed tree, several sphingolipid desaturases from crustaceans were used as outgroups. The resulting dataset was aligned with a MAFFT E-INS-i (*–maxiterate 1000 –genafpair*) [58]. Subsequently, trimming with a gap threshold of 95% was performed for each alignment (trimAl) [59]. Finally, next generation version of randomized axelerated maximum likelihood (RAxML-NG) [60] was performed in order to build the phylogenetic tree with LG + I + G4 as the best aa substitution model calculated by Modeltest-NG [61,62]. Confidence in the resulting phylogenetic tree branch topology was measured with all-in-one mode (*–all*). Auto bootstrapping (autoMRE, cutoff: 0.03) was converged after 350 replicates for the front-end desaturase tree, 650 replicates for the methyl-end desaturase tree and 450 replicates for the elongase tree.

### 2.3. Screening gammarid transcriptomes for ‘contaminant’ gene sequences from Rotifera symbionts

Since freshwater gammarids have often been reported to have bdelloid rotifers as epibionts [63–65], we analysed the occurrence of Rotifera in published transcriptomes from freshwater (*Gammarus pulex*, *Gammarus fossarum*, *Gammarus wautieri* and *Echinogammarus berilloni*) and marine (*Echinogammarus marinus*) wild caught gammarids [50]. We chose the dataset from Cogne *et al*. [50] not only because of its species coverage including both freshwater and marine species, but also because it is deposited as sequence read archive (SRA) enabling analysis and reproducibility of the raw sequencing data. Sequences retrieved from the above searches were introduced into a newly developed pipeline for the selection of the mitochondrial gene cytochrome c oxidase subunit 1 (*cox1*) to identify genetic contamination from the phylum Rotifera in the gammarid transcriptomes. Briefly, all the Metazoan complete mitochondrial (mtDNA) genomes available of NCBI (February of 2021) were retrieved and used as a reference to extract mitochondrial reads from the gammarid SRA, using the tool bbmap.sh and reformat.sh from BBMap v.37.77 (https://jgi.doe.gov/data-and-tools/software-tools/bbtools/bb-tools-user-guide/bbmap-guide/). The filtered mtDNA reads for each sample were assembled using rnaSPAdes v3.11.1 with default parameters [66]. Assembled contigs were blast searched against the nucleotide database of NCBI (NCBI-nt, Download; 24 August 2021), using the blastn from BLAST + v. 2.11.0 [67], and all contigs with hits with the phylum Rotifera retrieved. The mtDNA gene *cox1* is used as a standardized molecular identification system in most metazoan [68], thus one of the most sequenced genes available on NCBI. Therefore, to identify the putative Rotifera species present within each sample, all *cox1* sequences from Rotifera were retrieved from NCBI and used as a reference database for a blast search (blastn) to identify Rotifera *cox1* containing contigs. All hits that resulted in DNA sequences with identity scores higher than 75% were considered as positive for genetic contamination.

### 2.4. Detection of LC-PUFA biosynthesis genes in gammarids collected from the wild and laboratory cultures

In order to validate the occurrence of rotifer-origin genetic material in gammarid transcriptomes, we developed a polymerase chain reaction (PCR) assay to determine the presence of LC-PUFA biosynthetic gene sequences in *G. pulex* adults collected from the wild and laboratory cultures. The herein referred to as ‘wild-captured’ *G. pulex* sample consisted of one sole whole individual collected from a small freshwater stream in northern Germany (53°29'52″ N, 8°46'08″ E), which was preserved in RNAlater (Thermo Fisher Scientific, Waltham, MA, USA) until further analysis. The ‘laboratory cultured’ *G. pulex* sample consisted of one sole adult of an apparent same size as the wild-captured *G. pulex* sample, and derived from the second generation resulting from maintaining wild-captured *G. pulex* from the above location. The laboratory cultured *G. pulex* were maintained in fresh water at 18°C with coarse gravel as substrate and fed a mixed diet containing pellets made of dried carrot leaves, sugar beet and brewer grains. Furthermore, a second adult specimen of laboratory cultured *G. pulex* was dissected for collecting a hepatopancreas sample. Total RNA was extracted the three *G. pulex* samples (wild-captured, laboratory cultured and hepatopancreas) using the Illustra RNAspin Mini kit (Cytiva, Marlborough, MA, USA) following the manufacturer's instructions. The extracted RNA was treated with DNase I to avoid potential genomic DNA contamination. Subsequently, first strand complementary DNA (cDNA) was synthesized from 2 µg of total RNA using Moloney Murine Leukemia Virus Reverse Transcriptase (M-MLV RT) (Promega, Madison, WI, USA) and stored at −20°C until further analysis. Using the three cDNA samples (wild-captured whole individual, laboratory cultured whole individual, laboratory culture hepatopancreas) as templates, PCR (GoTaq®G2 Green Master Mix, Promega) were run using gene-specific primers targeting the sequences of *elovl2/5*, four front-end desaturases (*fed1-4*) and two methyl-end desaturases (*ωx1-2*) retrieved from *G. pulex* transcriptomes (electronic supplementary material, table S1). Moreover, a PCR with degenerate primers targeting a conserved region of the housekeeping gene *β-actin* (*actb*) for *Rotaria* species was also run to check for possible rotifer genetic material within the above three template cDNA samples (electronic supplementary material, table S1). Specific primers targeting the *G. pulex actb* were used for PCR as positive controls. Primer sequences and PCR conditions are indicated in electronic supplementary material, table S1. The resulting PCR products were purified on a 1% (w/v) agarose gel using the Wizard SV Gel and PCR Clean-Up System (Promega) and their identity confirmed by DNA sequencing (DNA Sequencing Service, IBMCP-UPV, Valencia, Spain) (electronic supplementary material, figure S1).

### 2.5. Sequence identity of the LC-PUFA biosynthesis genes among gammarid transcriptomes

The intra- and interspecific diversity of the LC-PUFA biosynthesis genes within gammarid transcriptomes was analysed by estimating the nucleotide (nt) identity scores (https://www.ebi.ac.uk/Tools/msa/clustalo/) as follows. First, we compared the elongase and desaturase nt sequences retrieved from transcriptomes of *Gammarus* species sampled at two geographical sites (intraspecific identity). The publicly available transcriptomes (table 1) enabled us to estimate the intraspecific identity for (1) *G. pulex*, comparing sequences occurring in transcriptomes built from samples collected in Germany (53°29'52″ N, 8°46'08″ E) and France (45°57'21″ N, 5°15'44″ E) (BioProject ID: PRJNA497972), and (2) *G. fossarum*, comparing sequences occurring in transcriptomes built from samples collected at two distinct French sites (47°53'31″ N, 6°58'53″ E, and 45°57'21″ N, 5°15'44″ E) (BioProject ID: PRJNA497972). Second, we determined the interspecific identity of the elongase and desaturase nt sequences retrieved from transcriptomes of two *Gammarus* species (*G. pulex* and *G. fossarum*) sampled at the same site, more specifically the Pollon River, Saint-Maurice de Rémens, France (45°57'21″ N, 5°15'44″ E) (BioProject ID: PRJNA497972). Finally, we analysed the sequence nt identity between two different gammarid species (*G. pulex* versus *G. lacustris*) sampled at very geographically distant locations, namely Germany (53°29'52″ N, 8°46'08″ E) and Russia (55°55'14.39″ N, 105°4'19.48″ E), respectively. Further details of the location and sample type can be found in table 1.

**Table 1.** Transcriptomic databases from gammarids mined for occurrence of key metabolic LC-PUFA genes (*elovl2/5*, *fed* and *ωx*). Details on species, geographical location of the sampling site and genes found (number in parentheses) are indicated. All databases were built from analysis of specimens collected from the wild except for *G. locusta* (PRJNA600472; laboratory culture), and correspond to whole individuals except for *G. pulex* hepatopancreas (PRJEB13055) and *G. fossarum* ‘internal tissues’ (PRJNA556212).

species name	BioProject ID	location	genes found
*Gammarus pulex*	PRJNA497972 [50]	Pollon River, Saint-Maurice de Rémens, France (45°57′21″N, 5°15′44″E)	*elovl*2/5, *fed* (4), *ωx* (2)
*Gammarus pulex*	PRJEB13055 [48]	Blanc-Gravier River, Liege, Belgium (50°34′60″N, 5°34′60″E)	None
*Gammarus pulex*	PRJEB13055 [48]	Vesdre River, Vaux-sous-Chevremont, Belgium (50°36′00″N, 5°37′58″E)	None
*Gammarus fossarum*	PRJNA497972 [50]	Pollon River, Saint-Maurice de Rémens, France (45°57′21″N, 5°15′44″E)	*elovl*2/5, *fed* (4), *ωx* (2)
*Gammarus fossarum*	PRJNA497972 [50]	Seebach River, Fellering, France (47°53′31″N, 6°58′53″E)	*elovl*2/5, *fed* (4), *ωx* (2)
*Gammarus fossarum*	PRJNA556212 [51]	Elgg, Switzerland (47°30′04.23″N, 8°51′09.40″ E)	*elovl*2/5, *fed* (2), *ωx* (1)
*Gammarus wautieri*	PRJNA497972 [50]	Galaveyson River, Le Grand Serre, France (45°16′27″N, 5°07′08″E)	*elovl*2/5, *fed* (4), *ωx* (2)
*Echinogammarus berilloni*	PRJNA497972 [50]	Saucats River, Saucats, France (44°39′34″N, 0°34′25″W)	*elovl*2/5, *fed* (4), *ωx* (2)
*Gammarus lacustris*	PRJNA660769 [53]	Lake no. 14, Baikal Lake, Irkutsk, Russia (51°55′14.39″N, 105°4′19.48″E)	*elovl*2/5, *fed* (2), *ωx* (2)
*Gammarus lacustris*	PRJNA505233 [55]	Lake no. 14, Baikal Lake, Irkutsk, Russia (51°55′14.39″N, 105°4′19.48″E)	*elovl*2/5, *fed* (2), *ωx* (2)
*Hyalellopsis costata*	PRJNA32160 [49]	Baikal, Bolshie Koty, across from ISU Biostation (51.90° N, 105.07° E)	*elovl*2/5, *fed* (2), *ωx* (2)
*Echinogammarus marinus*	PRJNA497972 [50]	sea coast, Portsmouth, UK (50°47′41″N, 1°01′50″W)	None
*Echinogammarus marinus*	PRJEB34316 [52]	intertidal mudflat, Saltash, UK (50°47′41″N, 1°01′50″W)	None
*Gammarus locusta*	PRJNA600472 [54]	south margin of the Sado estuary, Portugal (38°27′N, 08°43′W)	None

### 2.6. Genome location and exon–intron structure analysis of LC-PUFA biosynthesis genes in bdelloid rotifers

Expanding on the phylogeny results suggesting that sequences for LC-PUFA biosynthesis genes found in freshwater gammarid transcriptomes belong to Bdelloid rotifers, we identified the corresponding LC-PUFA biosynthesis gene sequences from *A. steineri* and studied their genome location (WGS Project: CAJNOJ01). In order to gain insight into the evolutionary origin of the LC-PUFA biosynthesis genes, we determined the exon–intron structure using the Exon-Intron Graphic Maker webpage (http://wormweb.org/exonintron, accessed 29 March 2023). Due to the exon–intron organizations of the *fed3* and *fed4* genes, as well as their unexpected location in the phylogenetic tree, we examined their genomic neighbourhood along the *A. steineri* genome in order to clarify whether these genes were not a spurious artefact of the genome assembly. We further addressed the evolutionary origin of the flanking genes with respect to the taxonomic distribution with blast (animal versus non-animal presence).

### 2.7. Functional characterization of LC-PUFA biosynthetic elongases and desaturases in yeast

Elongase and desaturase genes isolated from wild-captured *G. pulex* were functionally characterized by expressing their ORF in brewer's yeast *Saccharomyces cerevisiae*. Primers containing restriction sites for further cloning into the yeast expression vector pYES2 (Thermo Fisher Scientific) were used to amplify the ORF sequences using the high-fidelity polymerase Phusion Green High-Fidelity DNA Polymerase (Thermo Fisher Scientific) (electronic supplementary material, table S1). The PCR template consisted of cDNA prepared from total RNA of wild-captured *G. pulex* as described above. Each of the PCR products was digested with the corresponding restriction enzymes (electronic supplementary material, table S1) and further ligated into a similarly restricted pYES2 plasmid using T4 DNA ligase (Promega). The pYES2 constructs containing the ORF of the genes of interest (pYES2-Elovl25, pYES2-Fed1, pYES2-Fed2, pYES2Fed3, pYES2-Fed4, pYES2-ωx1 and pYES2-ωx2) were individually transformed into INV*Sc*1 *S. cerevisiae* competent cells (Thermo Fisher Scientific) using the *S.c.* EasyComp yeast transformation kit (Thermo Fisher Scientific). Selection and growth of recombinant yeast were carried out as described by Ribes-Navarro *et al*. [44]. To test the capacity of the elongase and desaturase enzymes, recombinant yeast expressing one sole LC-PUFA biosynthesis gene was supplemented with one of the following potential PUFA substrates selected according to LC-PUFA biosynthetic pathways (figure 1). For Elovl2/5, the assayed PUFA substrates included 18 : 3*n-*3, 18 : 2*n-*6, 18 : 4*n-*3, 18 : 3*n-*6, 20 : 5*n-*3, 20 : 4*n-*6, 22 : 5*n-*3, 22 : 4*n-*6 and 22 : 6*n-*3, whereas the exogenously PUFA supplied for transgenic yeast expressing front-end desaturases were 18 : 3*n-*3 and 18 : 2*n-*6 (Δ6 desaturation), 20 : 3*n-*3 and 20 : 2*n-*6 (Δ8 desaturation), 20 : 4*n-*3 and 20 : 3*n-*6 (Δ5 desaturation), and 22 : 5*n-*3 and 22 : 4*n-*6 (Δ4 desaturation). For methyl-end desaturases, the assayed PUFA included 18 : 2*n-*6, 18 : 3*n-*6, 20 : 2*n-*6, 20 : 3*n-*6, 20 : 4*n-*6, 22 : 4*n-*6 and 22 : 5*n-*6. Each PUFA substrate was supplemented as sodium salt at a final concentration of 0.5 mM (C_18_), 0.75 mM (C_20_), 1.0 mM (C_22_) in order to compensate for the decreased uptake efficiency as chain length increases. All fatty acid substrates (greater than 98–99% pure) used for the functional characterization assays were purchased from Nu-Chek Prep, Inc. (Elysian, MN, USA). Immediately after addition of the PUFA substrates, further supplementation of galactose (2%, w/v) induced gene expression. Along with their activity towards exogenously supplied PUFA substrates, we tested the ability of the two *ω*x to desaturate yeast endogenous fatty acid as previously reported [37,69]. For that purpose, transgenic yeast transformed with pYES2-ωx1 or pYES2-ωx2, as well as yeast transformed with the empty pYES2 vector (control), were grown in the absence of exogenously added PUFA substrates. Gene expression was induced with galactose as described above. After induction, yeast cultures were maintained at 30°C and under constant shaking (250 rpm) for 2 d until yeast were harvested by centrifugation at 1500*g* for 2 min. Yeast pellets were washed twice with 5 ml of ddH_2_O, homogenized in 6 ml of 2 : 1 (v/v) chloroform:methanol containing 0.01% (w/v) butylated hydroxytoluene (BHT, Sigma-Aldrich) as antioxidant and stored at −20°C for a minimum of 24 h in an oxygen-free atmosphere until further analysis. Yeast culture reagents including galactose, yeast nitrogen base without aa, raffinose, Tergitol NP-40, and uracil dropout medium were obtained from Sigma-Aldrich (St Louis, MO, USA).

### 2.8. Fatty acid analysis

The fatty acid composition of yeast collected from functional assays was determined using the method described by Monroig *et al*. [70]. Briefly, extracts of total lipids from yeast [71] were used to prepare fatty acid methyl esters (FAME) that were analysed using gas chromatography coupled with a flame ionization detector (GC-FID) [72]. The GC-FID was equipped with a fused silica 30 m × 0.25 mm open tubular column (TRACE TR-WAX, film thickness: 0.25 µm; Thermo Fisher Scientific) fitted with an on-column injection system and using helium as a carrier gas. Conversions of the LC-PUFA biosynthesizing enzymes towards the exogenously supplied PUFA substrates were calculated according to the formula [all product areas/(all product areas + substrate area)] × 100. Where necessary, further confirmation of FA products was carried out using an Agilent 6850 GC equipped with a mass spectrometry (MS) detector (5975 Series) and a 30 m × 0.25 mm open tubular column (DB5-MS, film thickness 0.25 µm; Agilent, Santa Clara, CA, USA) and comparing the spectra against those from the NIST library (MS Search v.2.0).

## 3. Results

### 3.1. Phylogeny of the LC-PUFA biosynthesis gene sequences retrieved from gammarid transcriptomes

**Figure 2.**
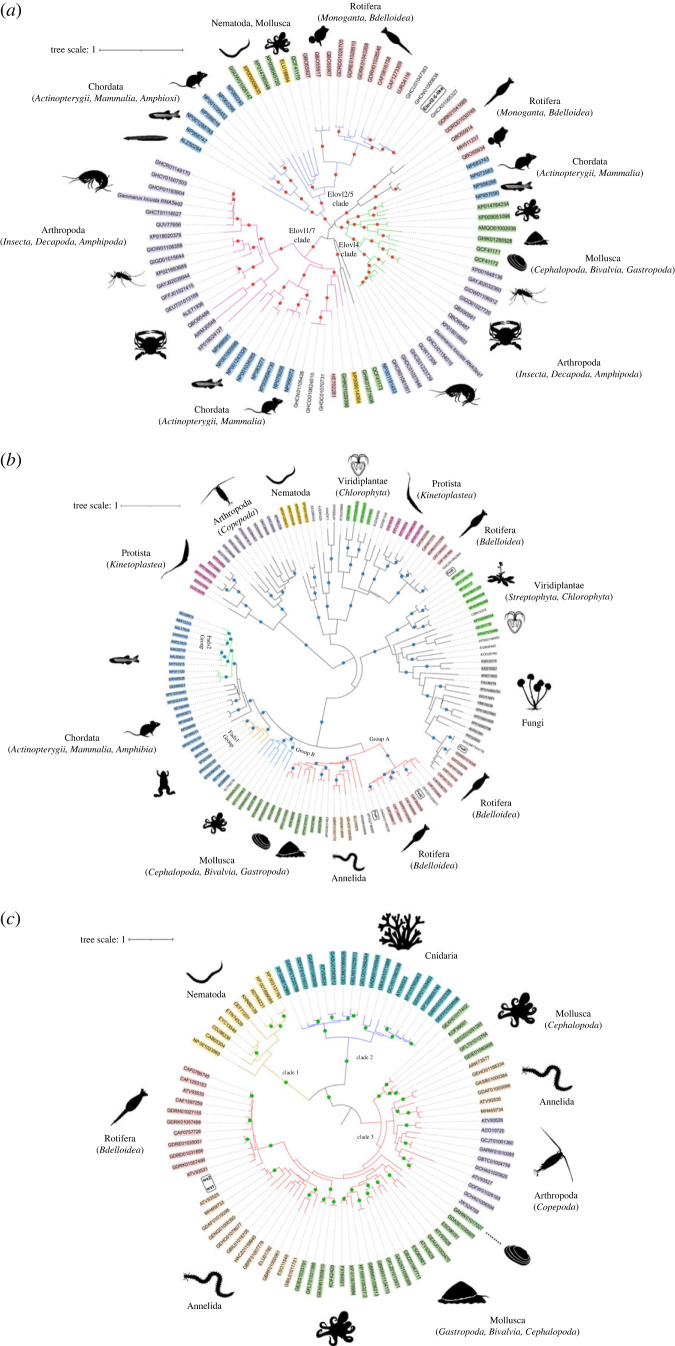
Condensed maximum likelihood phylogenetic trees of (*a*) elongases, (*b*) front-end desaturases, and (*c*) methyl-end desaturases. The protein evolutionary model was LG + I + G4. The tree was constructed using the maximum likelihood method by next generation version of randomized axelerated maximum likelihood (RAxML-NG). Confidence in the resulting phylogenetic tree branch topology was measured by auto-bootstrapping mode. Coloured dots on the nodes represent supporting values of 80% or above for RAxML. For each sequence, the accession numbers are given according to the information contained in the corresponding NCBI databases. Sequences subject to functional characterization in this study are rounded and highlighted in bold. Raw trees are available in the electronic supplementary material.

Full-length ORF sequences of putative PUFA elongases (*elovl2/5*), front-end desaturases and methyl-end desaturases could be only found in transcriptomes from freshwater gammarids but not marine species (table 1). Interestingly, no sequences encoding the above diagnostic LC-PUFA biosynthetic genes were found in the freshwater gammarid *G. pulex* transcriptome built using RNA extracted from hepatopancreas instead of whole individuals (PRJEB13055; table 1). Irrespective of the enzyme type, the phylogenetic analyses showed that the aa sequences retrieved from freshwater gammarid transcriptomes clustered closely (bootstrap > 90%) with their orthologues from rotifers, specifically from the Bdelloidea class (figure 2*a*; electronic supplementary material, figure S1). First, *elovl2/5* from the freshwater gammarids *G. pulex*, *G. fossarum*, *G. wautieri* and *E. berilloni* grouped together with putative *elovl2/5* from several *Rotaria* species, and more distantly with orthologues from other invertebrate groups including the functionally characterized Elovl2/5 from molluscs and amphioxus (figure 2*a*; electronic supplementary material, figure S1). Regarding front-end desaturases, the phylogenetic tree showed three well-differentiated clusters grouping all the gammarid sequences in two of these three clusters (figure 2*b*; electronic supplementary material, figure S2). The first cluster included two distinct sequences (Fed1 and Fed2) that could be consistently identified in transcriptomes from the freshwater gammarids analysed herein (figure 2*b*; electronic supplementary material, figure S2). These sequences clustered closely with front-end desaturase sequences belonging to the invertebrate Group A desaturases [73,74] and, more distantly, vertebrate sequences (figure 2*b*; electronic supplementary material, figure S2). Moreover, the first cluster included gammarid transcriptomic sequences termed as ‘Fed4’, which grouped along fatty acyl desaturases from several Fungi (figure 2*b*; electronic supplementary material, figure S2). The second cluster of front-end desaturases retrieved from gammarid transcriptomes (termed herein as ‘Fed3’) located closely to non-metazoan sequences from e.g. Kinetoplastea (figure 2*b*; electronic supplementary material, figure S2). Noteworthy, all Fed sequences retrieved from gammarid transcriptomes clustered closely to their rotifer orthologues. For methyl-end desaturases, all putative sequences retrieved from freshwater gammarid transcriptomes clustered within the so-called ‘Clade 3’ [12], along with methyl-end desaturases from Mollusca, Annelida, Arthropoda and Rotifera (figure 2*c*; electronic supplementary material, figure S3). Two distinct sequences termed herein as ‘*ωx1*’ and ‘*ωx2*’ were identified in each gammarid transcriptome from which methyl-end desaturases were retrieved (table 1). Importantly, the *ωx* sequences retrieved from freshwater gammarid transcriptomes grouped more closely to Rotifera sequences rather than other invertebrates including Arthropoda (figure 2*c*; electronic supplementary material, figure S3)

### 3.2. Occurrence of Rotifera mitochondrial genes in gammarid transcriptomes

Sequence homology searches of the *cox1* gene showed a very high identity with bdelloid rotifer *cox1* sequences (99.61% *Rotaria magnacalcarata*; 98.78% *Rotaria socialis*) within *G. pulex* RNASeq data (SRR8089720) (electronic supplementary material, table S2). Additionally, several other hits with a lower percentage of identity (79.4−86.6%) with other bdelloid rotifers were also identified. On the other hand, sequence homology searches of *cox1* within RNASeq from marine gammarids did not show any relevant identity with rotifer *cox1* sequences (electronic supplementary material, table S2).

### 3.3. Sequence identity of *elovl2/5*, *fed* and *ω*x retrieved from gammarid transcriptomes

The identity scores of the nt sequences from the LC-PUFA biosynthesis genes retrieved from transcriptomes of *Gammarus* species sampled at two geographical sites was determined to estimate the intraspecific diversity. The results showed that the identity scores of each homologous gene between *G. pulex* from Germany and France were all ≥ 96.9% (table 2). Similarly, comparison of sequences of each gene between *G. fossarum* from two distinct sites in France showed identity scores ranged between 96.9 and 99.5% (table 2). Subsequently, we analysed the interspecific identity of sequences encoding LC-PUFA biosynthetic enzymes from *G. fossarum* and *G. pulex* sampled at the same site in France (Pollon River, Saint-Maurice-de-Rémens). The results showed identity scores ranging between 98.7 and 99.9% (table 2). Finally, the results of the sequence comparison between *G. pulex* and *G. lacustris* sampled at very geographically distant locations (Germany and Russia, respectively) showed identity scores greater than 98% (table 2).

**Table 2.** Intra- and interspecific identity of the nucleotide sequences of selected LC-PUFA biosynthetic genes retrieved from transcriptomes of *Gammarus* spp. (*a*) Intraspecific comparisons made between sequences retrieved from *G. pulex* sampled in France (45°57′21″ N, 5°15′44″ E) and Germany (53°29'52″ N, 8°46'08″ E), and *G. fossarum* sampled at two French sites (47°53′31″ N, 6°58′53″ E and 45°57′21″ N, 5°15′44″ E). (*b*) Interspecific comparisons made between *G. pulex* and *G. fossarum* sampled at the same geographical location (Pollon River, Saint-Maurice de Rémens, France; 45°57′21″ N, 5°15′44″ E). (*c*) Interspecific comparisons made between *G. pulex* sampled in Germany (53°29'52″ N, 8°46'08″ E) and *G. lacustris* sampled in Russia (Lake no. 14, Baikal Lake, Irkutsk, 51°55′14.39″ N, 105°4′19.48″ E). *No gene found in the *G. lacustris* transcriptome (PRJNA660769), as indicated in table 1.

	% identity						
	*elovl*2/5	*fed*1	*fed*2	*fed*3	*fed*4	*ωx*1	*ωx*2
(*a*) intraspecific							
*Gammarus pulex*	99.6	99.9	98.4	99.8	97.3	99.6	99.7
*Gammarus fossarum*	97.9	96.9	98.5	99.5	97.5	98.2	99.5
(*b*) interspecific							
*G. pulex* versus *G. fossarum*	99.6	99.5	98.7	99.8	99.9	99.7	99.8
(*c*) interspecific							
*G. pulex* versus *G. lacustris*	98.1	99.7	99.4	*	*	99.5	99.5

### 3.4. Occurrence of LC-PUFA biosynthesis genes in gammarids collected from the wild and laboratory cultures

**Figure 3.**
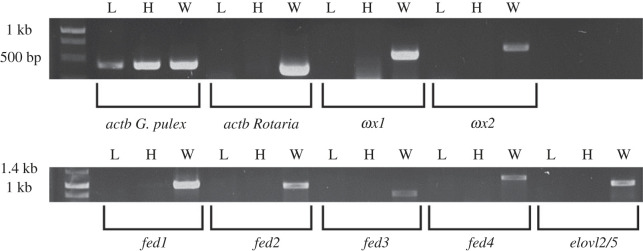
RT-PCR assays for detection of candidate genes (*ωx1*, *ωx2*, *fed1*, *fed2*, *fed3*, *fed4*, and *elovl2/5*) and housekeeping genes (*actb* from *G. pulex* and *Rotaria*) in laboratory cultured whole individual *G. pulex* (L), hepatopancreas (H) and wild caught whole individual *G. pulex* (W).

We next confirmed the occurrence of rotifer genetic material in freshwater gammarids by detecting the *in silico* retrieved LC-PUFA biosynthesis genes by RT-PCR on cDNA prepared from laboratory cultured whole individual adult *G. pulex* (L), hepatopancreas from adult *G. pulex* (H), wild-captured adult *G. pulex* (W) (figure 3). RT-PCR targeting each of the candidate LC-PUFA biosynthetic genes were positive for W, but not L and H (figure 3). Similarly, only the W sample, but not L and H, showed a positive band when targeting the *Rotaria actb* chosen as diagnostic gene to detect the presence of epibiotic rotifers (figure 3; electronic supplementary material, figure S4). Moreover, sequencing results for the *Rotaria actb* band confirmed its identity as a rotifer gene. A positive control targeting the *G. pulex actb* was positive in all three cDNA samples (L, H and W) (figure 3).

### 3.5. Exon–intron organization of LC-PUFA biosynthesis genes from Bdelloidea rotifers

**Figure 4.**
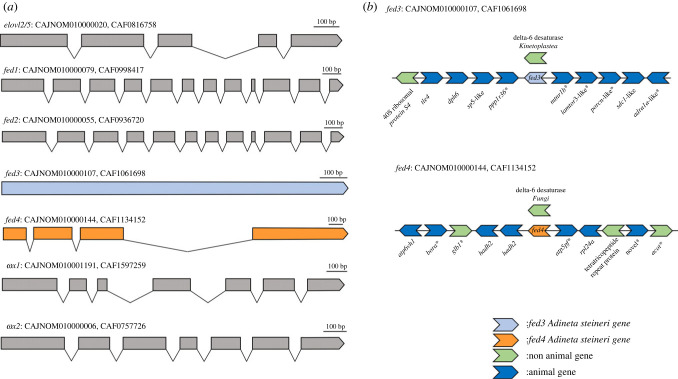
Exon–intron organization and genomic neighbourhood of LC-PUFA biosynthetic genes from *Adineta steineri*. (*a*) Exon–intron structure of candidate LC-PUFA biosynthetic genes (*elovl2/5*, *fed1*, *fed2*, *fed3*, *fed4*, *ωx1*, and *ωx2*) in the *A. steineri* genome. Introns are indicated by solid lines between exons (coloured boxes). All genes analysed have introns except for *fed3*, which is intronless. Besides, *fed4* has an exon–intron organization consistent with its HGT origin. (*b*) Distribution of the *fed3* and *fed4* genes across the *A. steineri* genome. Both genes are flanked by animal genes in the genome according to identity analyses results (BLAST). Moreover, BLAST results also revealed that *fed3* has a protist origin whereas *fed4* has fungi origin. *Identity scores less than 45%.

**Figure 5.**
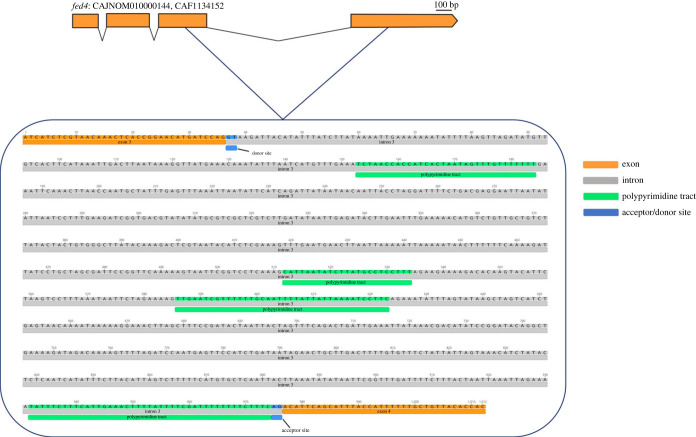
Nucleotide sequence of the *Adineta steineri fed4* genomic sequence. The *A. steineri fed4* genomic sequence (CAJNOM010000144, CAF1134152) has characteristic motifs of an introner-like sequence (ILE). Intron 3 (grey) is flanked by exons 3 and 4 (orange). The particular nucleotide sequences typical from regular spliceseosomal introns (RSI) are polypyrimidine tracts (green) and acceptor/donor sites (blue) for the spliceosome recognition and removal [75,97].

To confirm the results from the phylogenetic analysis suggesting that the LC-PUFA biosynthesis genes retrieved from gammarid transcriptomes belong to epibiotic bdelloid rotifers, we mapped them into the genome of *A. steineri* (WGS project: CAJNOM01; Assembly: GCA_905250015.1) and determined their exon–intron organization (figure 4*a*). Our results revealed that the seven LC-PUFA biosynthesis genes retrieved from gammarid transcriptomes, namely one elongase (*elovl2/5*), four *fed*, and two *ωx*, are genome-anchored in *A. steineri*. The analysis of their exon–intron organization showed that the front-end desaturase herein termed ‘*fed3*’ is an intronless gene (figure 4*a*). Besides, the *fed4* sequence contained characteristic features of ‘introner-like’ elements [75], including a relatively long intron (third, 940 bp), conserved motifs with pyrimidine rich regions, and acceptor and donor sites for recognition by the spliceosome (figure 5). The particular exon–intron organization of *fed3* and *fed4*, along with their close phylogenetic relationship with sequences from kinetoplastids and fungi, respectively, suggested HGT as the evolutionary origin of these genes in bdelloid rotifers. To exclude the possibility that such kinetoplastid and fungi sequences might be a contamination in bdelloids, we further examined the genomic neighbourhood of both *fed3* and *fed4* along the *A. steineri* genome (figure 4*b*). The *A. steineri fed3* and *fed4* are both flanked by genes that have orthologues in other metazoan species, confirming that both *fed3* and *fed4* are real components of the *A. steineri* genome and not a putative contamination of kinetoplastids and fungi (figure 4*b*).

### 3.6. Functional characterization of LC-PUFA biosynthesis enzymes from bdelloid rotifers

The activities of the LC-PUFA biosynthetic enzymes encoded by the diagnostic genes from bdelloid rotifers were investigated by expressing their ORF in yeast. Transgenic yeasts expressing the *elovl2/5* were able to elongate both C_18_ and C_20_ PUFA substrates, but not C_22_, to their corresponding C_20_ and C_22_ elongation products, respectively (electronic supplementary material, table S3). Regarding front-end desaturases, Fed1 showed Δ5 desaturase activity as denoted by the conversions of 20 : 4*n-*3 and 20 : 3*n-*6 into 20 : 5*n-*3 (EPA) and 20 : 4*n-*6 (ARA), respectively (electronic supplementary material, table S4). Functional characterization of Fed3 showed this enzyme is a Δ6 desaturase as it was able to convert 18 : 3*n-*3 and 18 : 2*n-*6 into 18 : 4*n-*3 and 18 : 3*n-*6, respectively (electronic supplementary material, table S4). Fed2 and Fed4 did not show activity toward any of the assayed substrates (data not shown). For methyl-end desaturases, both characterized enzymes showed dual Δ12 and Δ15 activities as they were able to desaturate 18 : 1*n-*9 to 18 : 2*n-*6 (Δ12 desaturation), as well as 18 : 2*n-*6 and 18 : 3*n-*6 to 18 : 3*n-*3 and 18 : 4*n-*3, respectively (Δ15 desaturations) (electronic supplementary material, table S5).

## 4. Discussion

Our sequence retrieval strategy has revealed a markedly different repertoire of LC-PUFA biosynthetic genes in transcriptomes from freshwater gammarids compared to marine species, with the former having a generally conserved number of *elovl2/5* (1 gene), *fed* (4 genes) and *ωx* (2 genes), and none in the latter. Instances of animal lineages with enhanced LC-PUFA biosynthetic capacity in species inhabiting freshwater ecosystems compared to that of marine counterparts have been previously reported in sticklebacks and American soles [76,77]. However, such an evolutionary innovation enabling colonization of freshwater environments by these teleosts occurred in one specific front-end desaturase gene, *fads2* [76,77], rather than an acquisition of multiple genes as suggested by the gammarid transcriptomic database searches. The present study provides compelling evidence demonstrating that the unexpected occurrence of key desaturase and elongase genes in freshwater gammarid transcriptomes, rather than gammarids themselves, is accounted for by the presence of bdelloid rotifers in the analysed samples.

Our phylogenetic analysis revealed that, with the exception of the herein termed *fed3* and *fed4*, the LC-PUFA biosynthetic genes used as diagnostic markers clustered together with orthologues from bdelloid rotifers, particularly *Rotaria* and *Adineta* species. These results strongly suggested that multiple transcriptomes from freshwater gammarids contain rotifers’ genetic material contaminating those publicly available databases. Due to the fact that bdelloid rotifers are typical epibionts of freshwater gammarids associated with their branchial plates and appendages (e.g. 22 different species reported in *G. pulex*), their high prevalence (up to 80%), and their relatively small size (ranging from 150 to 700 μm approx.) [63–65,78], it is reasonable to speculate that the gammarid specimens used for RNA sequencing contained rotifers that were not removed prior to analysis. As opposed to whole individuals [50], the transcriptome built from *G. pulex* hepatopancreas [48] did not result in any positive hit against any of the LC-PUFA biosynthesis gene markers assessed, confirming that internal body parts of the gammarid are free from epibiotic rotifers. Unlike freshwater species, marine gammarids such as *E. marinus* [50,52] and *G. locusta* [54] did not have any of the diagnostic LC-PUFA biosynthesis genes, consistent with the fact that the above described epibiotic relationship between gammarids (host) and bdelloid rotifers (epibiont) is restricted to freshwater environments [64]. Our data further support that the abundance of rotifers in the gammarid samples used for RNA sequencing was not negligible according to the presence of a remarkable amount of rotifer-derived mitochondrial genes (*cox*) in some of the available RNA sequencing data [50]. It is also worth noting that, while pipelines for de novo transcriptome assembly typically imply filtering strategies [51,79–81], these appear not have been effective in removing rotifer contamination from the analysis. This probably explains why, despite not being the targeted species (gammarid), full-length sequences for all diagnostic LC-PUFA biosynthesis gene markers were found in most freshwater gammarid transcriptomes when such genomic platforms were built with freshly sampled wild-captured whole individuals. Laboratory cultured gammarids that have progressively lost epibionts after successive generations and/or moulting events, as well as internal tissues such as the abovementioned hepatopancreas [48], arise as safe, clean alternative samples, as supported by the PCR-based determinations of gene markers in *G. pulex*. As noted above, our phylogenetic analyses could only identify *Rotaria* and less probably *Adineta* as the most likely bdelloid genera contributing to the occurrence of LC-PUFA biosynthetic gene sequences in gammarid transcriptomes. Interestingly, only slight variations along the nucleotide sequences of the analysed genes were found, even when comparing sequences from samples collected from very distantly located sites like Central Europe (France, Belgium, Germany and Switzerland) and Baikal Lake (Russia). These results show that all epibiotic rotifers inhabiting the exoskeleton of freshwater gammarids are very closely related irrespectively of their geographical location and biotope.

Phylogenetics suggested that Fed3 and Fed4 are from a heterologous origin unlike other LC-PUFA biosynthesis genes investigated herein, since they clustered together with front-end desaturases from kinetoplastids (protists) and fungi, respectively. To clarify this point, we examined the genomic location of *fed3* and *fed4* within the genome of the bdelloid rotifer *A. steineri* [82], demonstrating both genes are genome-anchored. Similarly, other LC-PUFA biosynthetic genes, namely *elovl2/5*, *fed1*, *fed2*, *ωx1* and *ωx2*, were also confirmed to be genome-anchored, demonstrating that the complement of LC-PUFA biosynthetic genes in bdelloid rotifers goes well beyond *ωx1* and *ωx2* previously reported in *Adineta vaga* [12]. Importantly, in agreement with their unexpected location in the phylogenetic trees, we established that *fed3* and *fed4* likely originate via HGT, a well-documented evolutionary mechanism by which bdelloid rotifers have gained bacterial, protist and fungal genes [57,83–87]. Moreover, analysis of the exon–intron organization in *fed3* and *fed4* further supported these were likely instances of HGT. The *A. steineri fed3* is intronless, characteristic of horizontally transferred genes by RNA retrotransposition [88–90] likely from kinetoplastids that are often prevalent in the freshwater habitats occupied by bdelloid rotifers [91–94]. Unlike *fed3*, *fed4* contains introns suggesting a relatively more complex mechanism by which *fed4* has been transferred into the *A. steineri* genome, combining RNA retrotransposition as described for *fed3* and/or further intron gain likely occurring via the intron-generating transposons called ‘introners’ [95,96]. Consistently, the structure of the third intron of the *A. steineri fed4* includes typical features of ‘introner-like’ elements [75], particularly those belonging to the ‘novel RNA-propagated elements' family, which have been described in fungi [97]. Introners had been originally described in the picoeukaryote *Micromonas* [96], the pelagic tunicate *Oikopleura dioica* [98], and the dothideomycete fungus *Meloidogyne graminicola* [99]. However, it has been recently discovered that they are more widespread in eukaryotes than originally anticipated and, interestingly, disproportionately common in aquatic lineages [97]. While fungi, identified as potential donors justifying the origin of *fed4*, have been previously reported to explain HGT to bdelloid rotifers [83,87], no previous studies have reported HGT to bdelloid rotifers from kinetoplastids as suggested herein for *fed3*. However, abundance of kinetoplastids in aquatic ecosystems, particularly freshwater environments, makes them likely donors in a HGT of *fed3* to rotifers [92,93,100,101]. In agreement, the substrate specificity of kinetoplastid front-end desaturases shared features with that of the bdelloid rotifer Fed3-like desaturase characterized in our study, further supporing a common origin.

The bdelloid rotifer Fed3 was functionally characterized as a Δ6 desaturase and showed no activity as Δ8 desaturase. These results are in agreement with functions reported in one of the three front-end desaturases from the kinetoplastid *Leishmania major*, which was shown to have Δ6 activity but no Δ8 [102]. Such regioselectivity is markedly different from that of multiple animal front-end desaturases characterized from mammals [103], teleosts [104] and copepods [37] that, in addition to Δ6, have also Δ8 desaturase activity. Exceptions to the above pattern can be found in, for example, the bivalve mollusc *Sinonovacula constricta* Δ6 Fed [105], although this protein is phylogenetically unrelated to those from the cluster formed by kinetoplastid and bdelloid rotifer Fed3 [10]. A more widespread regioselectivity among invertebrates’ Fed is Δ5 desaturase [10], herein demonstrated to occur in the bdelloid rotifer Fed1 but not Fed2 despite both genes being duplicates as our phylogenetics and exon–intron organization analyses in *A. steineri* clearly demonstrated. Consequently, since our yeast system did not show any detectable activity that would suggest neofunctionalization beyond Δ5 activity, it can be hypothesized that functional redundancy has driven loss of function in Fed2 [106,107]. Reasons accounting for the loss of function in Fed4 are less clear, although they could be related to their HGT origin as reported elsewhere in the literature [108,109]. Along the functions of front-end desaturases, characterization of the two methyl-end desaturases (*ω* × 1 and *ω* × 2) and Elovl2/5 demonstrate that bdelloid rotifers have complete enzymatic capacity that enable them to biosynthesize de novo LC-PUFA up to ARA and EPA (figure 1). Indeed, both *ω* × 1 and *ω* × 2 from bdelloid rotifers have Δ12 and Δ15 desaturase activities, enabling the conversion of OA to LA and, subsequently, ALA (figure 1). While missing the Δ17 activity also contained in the *A. vaga* methyl-end desaturases [12], the combined Δ12/Δ15 regioselectivities within the same enzyme demonstrated herein for both *ω*x desaturase enzymes is consistent with their bdelloid rotifer origin and contrasts that from non-rotifer methyl-end desaturases in which the gene complement typically includes also an *ω*3 desaturase [10]. Elovl2/5 exhibited activity towards C_18_ and C_20_ PUFA substrates, converting them into the corresponding C_20_ and C_22_ products, respectively. Such elongation abilities by Elovl2/5 account for all the elongations required to biosynthesize ARA and EPA (figure 1). However, DHA biosynthesis capacity in bdelloid rotifers appears to be limited in the apparent absence of a Δ4 front-end desaturase as our results suggest (figure 1). Potential DHA synthesis in bdelloid rotifers, if existing, would necessarily proceed via the so-called Sprecher pathway but, additionally to Elovl2/5, participation of further PUFA elongases with the ability to elongate 22 : 5*n-*3 to 24 : 5*n-*3 would be mandatory (figure 1). Importantly, the above results raise the question of whether bdelloid rotifers can supply their hosts (gammarids) with physiologically essential LC-PUFA originated via biosynthesis, which might be highly advantageous for the gammarids according to their limited biosynthetic capacity [44] and the relatively low availability of preformed LC-PUFA in freshwater environments [6,110].

In conclusion, the present study demonstrates that multiple transcriptomes from freshwater gammarids contain sequences that can be unequivocally assigned to bdelloid rotifers, well-established epibionts of gammarids. More specifically, we determined the systematic occurrence of key metabolic enzymes of LC-PUFA biosynthesis, and clarified that such occurrence is independent from the species and their geographical origin when transcriptomes are built from freshly sampled wild-captured whole individuals. Genomic location and exon–intron organization further demonstrated that the diagnostic elongase and desaturase genes are bona fide components of the *A. steineri* (and presumably other bdelloid rotifers) genome. Moreover, we provide compelling evidence demonstrating that two of the front-end desaturase genes, namely *fed3* and *fed4*, have been acquired via HGT. Our findings offer a critical example of the value of functional genomics and critical assessment of sequence data to infer ecological impacts in the age of *Omics*.

## Data Availability

All data produced in the present study are presented in the paper and associated electronic supplementary material. The data are provided in electronic supplementary material [111].

## References

[1] Tocher DR. 2015 Omega-3 long-chain polyunsaturated fatty acids and aquaculture in perspective. Aquaculture **449**, 94-107. (10.1016/j.aquaculture.2015.01.010)

[2] Bazinet R, Layé S. 2014 Polyunsaturated fatty acids and their metabolites in brain function and disease. Nat. Rev. Neurosci. **15**, 771-785. (10.1038/nrn3820) 25387473

[3] Innis SM. 2007 Dietary (*n-*3) fatty acids and brain development. J. Nutr. **137**, 855-859. (10.1093/jn/137.4.855) 17374644

[4] Závorka L et al. 2023 The role of vital dietary biomolecules in eco-evo-devo dynamics. Trends Ecol. Evol. **38**, 72-84. (10.1016/j.tree.2022.08.010) 36182405

[5] Swanson D, Block R, Mousa SA. 2012 Omega-3 fatty acids EPA and DHA: health benefits throughout life. Adv. Nutr. **3**, 1-7. (10.3945/an.111.000893) 22332096 PMC3262608

[6] Twining CW et al. 2021 The evolutionary ecology of fatty-acid variation: Implications for consumer adaptation and diversification. Ecol. Lett. **24**, 1709-1731. (10.1111/ele.13771) 34114320

[7] Nichols DS. 2003 Prokaryotes and the input of polyunsaturated fatty acids to the marine food web. FEMS Microbiol. Lett. **219**, 1-7. (10.1016/S0378-1097(02)01200-4) 12594015

[8] Khozin-Goldberg I, Iskandarov U, Cohen Z. 2011 LC-PUFA from photosynthetic microalgae: occurrence, biosynthesis, and prospects in biotechnology. Appl. Microbiol. Biotechnol. **91**, 905-915. (10.1007/s00253-011-3441-x) 21720821

[9] Pereira SL, Leonard AE, Mukerji P. 2003 Recent advances in the study of fatty acid desaturases from animals and lower eukaryotes. Prostaglandins Leukot. Essent. Fat. Acids **68**, 97-106. (10.1016/S0952-3278(02)00259-4) 12538073

[10] Monroig Ó, Shu-Chien AC, Kabeya N, Tocher DR, Castro LFC. 2022 Desaturases and elongases involved in long-chain polyunsaturated fatty acid biosynthesis in aquatic animals: from genes to functions. Prog. Lipid Res. **86**, 101157. (10.1016/j.plipres.2022.101157) 35104467

[11] Monroig Ó, Kabeya N. 2018 Desaturases and elongases involved in polyunsaturated fatty acid biosynthesis in aquatic invertebrates: a comprehensive review. Fish. Sci. **84**, 911-928. (10.1007/s12562-018-1254-x)

[12] Kabeya N, Fonseca MM, Ferrier DEK, Navarro JC, Bay LK, Francis DS, Tocher DR, Castro LFC, Monroig Ó. 2018 Genes for *de novo* biosynthesis of ω-3 polyunsaturated fatty acids are widespread in animals. Sci. Adv **4**, eaar6849. (10.1126/sciadv.aar6849) 29732410 PMC5931762

[13] Menzel R, Von Chrzanowski H, Tonat T, Van Riswyck K, Schliesser P, Ruess L. 2019 Presence or absence? Primary structure, regioselectivity and evolution of Δ12/*ω*3 fatty acid desaturases in nematodes. Biochim. Biophys. Acta **1864**, 1194-1205. (10.1016/j.bbalip.2019.05.001) 31108204

[14] Holm HC, Fredricks HF, Bent SM, Lowenstein DP, Ossolinski JE, Becker KW, Johnson WM, Schrage K, Van Mooy BAS. 2022 Global ocean lipidomes show a universal relationship between temperature and lipid unsaturation. Science **376**, 1487-1491. (10.1126/science.abn7455) 35737766

[15] Hixson SM, Arts MT. 2016. Climate warming is predicted to reduce omega-3, long-chain, polyunsaturated fatty acid production in phytoplankton. Glob. Change Biol. **22**, 2744-2755. (10.1111/gcb.13295) 27070119

[16] Jovanovic S, Dietrich D, Becker J, Kohlstedt M, Wittmann C. 2021 Microbial production of polyunsaturated fatty acids—high-value ingredients for aquafeed, superfoods, and pharmaceuticals. Curr. Opin. Biotechnol. **69**, 199-211. (10.1016/j.copbio.2021.01.009) 33540327

[17] Metz JG et al. 2001 Production of polyunsaturated fatty acids by polyketide synthases in both prokaryotes and eukaryotes. Science **293**, 290-293. (10.1126/science.1059593) 11452122

[18] Castro LFC, Wilson JM, Gonçalves O, Galante-Oliveira S, Rocha E, Cunha I. 2011 The evolutionary history of the stearoyl-CoA desaturase gene family in vertebrates. BMC Evol. Biol. **11**, 132. (10.1186/1471-2148-11-132) 21595943 PMC3112091

[19] Haritos VS, Horne I, Damcevski K, Glover K, Gibb N, Okada S, Hamberg M. 2012 The convergent evolution of defensive polyacetylenic fatty acid biosynthesis genes in soldier beetles. Nat. Commun. **3**, 1150. (10.1038/ncomms2147) 23093187

[20] Zhou XR, Horne I, Damcevski K, Haritos V, Green A, Singh S. 2008 Isolation and functional characterization of two independently-evolved fatty acid Δ12-desaturase genes from insects. Insect Mol. Biol. **17**, 667-676. (10.1111/j.1365-2583.2008.00841.x) 19133076

[21] Semmelmann F et al. 2019 Functional characterisation of two Δ12-desaturases demonstrates targeted production of linoleic acid as pheromone precursor in *Nasonia*. J. Exp. Biol. **222**, jeb201038. (10.1242/jeb.201038) 31019064

[22] Haritos VS, Horne I, Damcevski K, Glover K, Gibb N. 2014 Unexpected functional diversity in the fatty acid desaturases of the flour beetle *Tribolium castaneum* and identification of key residues determining activity. Insect Biochem. Mol. Biol. **51**, 62-70. (10.1016/j.ibmb.2014.05.006) 24880119

[23] Castro LFC, Tocher DR, Monroig Ó. 2016 Long-chain polyunsaturated fatty acid biosynthesis in chordates: insights into the evolution of Fads and Elovl gene repertoire. Prog. Lipid Res. **62**, 25-40. (10.1016/j.plipres.2016.01.001) 26769304

[24] Sperling P, Ternes P, Zank TK, Heinz E. 2003 The evolution of desaturases. Prostaglandins Leukot. Essent. Fat. Acids **68**, 73-95. (10.1016/S0952-3278(02)00258-2) 12538072

[25] Titocci J, Fink P. 2022 Food quality impacts on reproductive traits, development and fatty acid composition of the freshwater calanoid copepod *Eudiaptomus* sp. J. Plankton Res. **44**, 528-541. (10.1093/plankt/fbac030)

[26] De Troch M, Boeckx P, Cnudde C, Van Gansbeke D, Vanreusel A, Vincx M, Caramujo MJ. 2012 Bioconversion of fatty acids at the basis of marine food webs: insights from a compound-specific stable isotope analysis. Mar. Ecol. Prog. Ser. **465**, 53-67. (10.3354/meps09920)

[27] Helenius L, Budge SM, Johnson CL. 2020 Stable isotope labeling reveals patterns in essential fatty acid growth efficiency in a lipid-poor coastal calanoid copepod. Mar. Biol. **167**, 178. (10.1007/s00227-020-03794-8)

[28] Bell MV, Dick JR, Anderson TR, Pond DW. 2007 Application of liposome and stable isotope tracer techniques to study polyunsaturated fatty acid biosynthesis in marine zooplankton. J. Plankton Res. **29**, 417-422. (10.1093/plankt/fbm025)

[29] Pérez JA, Reis DB, Ramírez D, Acosta NG, Dorta-Guerra R, Jerez S, Rodríguez C. 2022 *In vivo* biosynthesis of long-chain polyunsaturated fatty acids by the euryhaline rotifer (*Brachionus plicatilis*). Aquaculture **560**, 738415. (10.1016/j.aquaculture.2022.738415)

[30] Pairohakul S. 2013 *Evidence for polyunsaturated fatty acid biosynthesis in the ragworm (Nereis virens) and the lugworm (Arenicola marina)*. Newcastle, UK: School of Marine Science, University of Newcastle Upon Tyne. See https://theses.ncl.ac.uk/jspui/handle/10443/2295.

[31] Schauer PS, Simpson KL. 1985 Bioaccumulation and bioconversion of dietary labeled fatty acids in Artemia and Winter Flounder (*Pseudopleuronectes americanus*). Can. J. Fish. Aquat. Sci. **42**, 1430-1438. (10.1139/f85-179)

[32] Jøstensen JP, Landfald B. 1997 High prevalence of polyunsaturated-fatty-acid producing bacteria in arctic invertebrates. FEMS Microbiol. Lett. **151**, 95-101. (10.1016/S0378-1097(97)00148-1)

[33] Tan K, Zheng H. 2022 Endogenous LC-PUFA biosynthesis capability in commercially important mollusks. Crit. Rev. Food Sci. Nutr. **62**, 2836-2844. (10.1080/10408398.2020.1860896) 33354986

[34] Ting SY, Janaranjani M, Merosha P, Sam K-K, Wong SC, Goh P-T, Mah M-Q, Kuah M-K, Chong Shu-Chien A. 2020 Two elongases, Elovl4 and Elovl6, fulfill the elongation routes of the LC-PUFA biosynthesis pathway in the orange mud crab (*Scylla olivacea)*. J. Agric. Food Chem. **68**, 4116-4130. (10.1021/acs.jafc.9b06692) 32186869

[35] Mah MQ, Kuah MK, Ting SY, Merosha P, Janaranjani M, Goh PT, Jaya-Ram A, Shu-Chien AC. 2019 Molecular cloning, phylogenetic analysis and functional characterisation of an Elovl7-like elongase from a marine crustacean, the orange mud crab (*Scylla olivacea*). Comp. Biochem. Physiol. B Biochem. Mol. Biol. **232**, 60-71. (10.1016/j.cbpb.2019.01.011) 30831207

[36] Sun P, Zhou Q, Monroig Ó, Navarro JC, Jin M, Yuan Y, Wang X, Jiao L. 2020 Cloning and functional characterization of an *elovl4*-like gene involved in the biosynthesis of long-chain polyunsaturated fatty acids in the swimming crab *Portunus trituberculatus*. Comp. Biochem. Physiol. B Biochem. Mol. Biol. **242**, 110408. (10.1016/j.cbpb.2020.110408) 31958500

[37] Kabeya N, Ogino M, Ushio H, Haga Y, Satoh S, Navarro JC, Monroig Ó. 2021 A complete enzymatic capacity for biosynthesis of docosahexaenoic acid (DHA, 22:6*n-*3) exists in the marine Harpacticoida copepod *Tigriopus californicus*. Open Biol. **11**, 200402. (10.1098/rsob.200402) 33906414 PMC8080000

[38] Ting SY et al. 2022 Long-chain polyunsaturated fatty acid biosynthesis in a land-crab with advanced terrestrial adaptations: molecular cloning and functional characterization of two fatty acyl elongases. Comp. Biochem. Physiol. B Biochem. Mol. Biol. **262**, 110773. (10.1016/j.cbpb.2022.110773) 35718326

[39] Ramos-Llorens M, Ribes-Navarro A, Navarro JC, Hontoria F, Kabeya N, Monroig Ó. 2023 Can *Artemia franciscana* produce essential fatty acids? Unveiling the capacity of brine shrimp to biosynthesise long-chain polyunsaturated fatty acids. Aquaculture **563**, 738869. (10.1016/j.aquaculture.2022.738869)

[40] Boyen J, Ribes-Navarro A, Kabeya N, Monroig Ó, Rigaux A, Fink P, Hablützel P, Navarro JC, De Troch M. 2023 Functional characterization reveals a diverse array of metazoan fatty acid biosynthesis genes. Mol. Ecol. **32**, 970-982. (10.1111/mec.16808) 36461663

[41] Nielsen BLH, Gøtterup L, Jørgensen TS, Hansen BW, Hansen LH, Mortensen J, Jepsen PM. 2019 *n-*3 PUFA biosynthesis by the copepod *Apocyclops royi* documented using fatty acid profile analysis and gene expression analysis. Biol. Open **8**, bio038331. (10.1242/bio.038331) 30723075 PMC6398464

[42] Lin Z, Hao M, Zhu D, Li S, Wen X. 2017 Molecular cloning, mRNA expression and nutritional regulation of a Δ6 fatty acyl desaturase-like gene of mud crab, *Scylla paramamosain*. Comp. Biochem. Physiol. B Biochem. Mol. Biol. **208**, 29-37. (10.1016/j.cbpb.2017.03.004) 28373120

[43] Lin Z, Hao M, Huang Y, Zou W, Rong H, Wen X. 2018 Cloning, tissue distribution and nutritional regulation of a fatty acyl Elovl4-like elongase in mud crab, *Scylla paramamosain* (Estampador, 1949). Comp. Biochem. Physiol. B Biochem. Mol. Biol. **217**, 70-78. (10.1016/j.cbpb.2017.12.010) 29277642

[44] Ribes-Navarro A, Navarro JC, Hontoria F, Kabeya N, Standal IB, Evjemo JO, Monroig Ó. 2021 Biosynthesis of long-chain polyunsaturated fatty acids in marine gammarids: molecular cloning and functional characterisation of three fatty acyl elongases. Mar. Drugs **19**, 226. (10.3390/MD19040226) 33923820 PMC8073319

[45] Monroig Ó, Guinot D, Hontoria F, Tocher DR, Navarro JC. 2012 Biosynthesis of essential fatty acids in *Octopus vulgaris* (Cuvier, 1797): molecular cloning, functional characterisation and tissue distribution of a fatty acyl elongase. Aquaculture **360**, 45-53. (10.1016/j.aquaculture.2012.07.016)

[46] Ran Z et al. 2019 Biosynthesis of long-chain polyunsaturated fatty acids in the razor clam *Sinonovacula constricta*: characterization of four fatty acyl elongases and a novel desaturase capacity. Biochim. Biophys. Acta **1864**, 1083-1090. (10.1016/j.bbalip.2019.04.004) 31002943

[47] Liu H, Zhang H, Zheng H, Wang S, Guo Z, Zhang G. 2014 PUFA biosynthesis pathway in marine scallop *Chlamys nobilis* Reeve. J. Agric. Food Chem. **62**, 12 384-12 391. (10.1021/jf504648f) 25439983

[48] Gismondi E, Thomé JP. 2016 Transcriptome of the freshwater amphipod *Gammarus pulex* hepatopancreas. Genom. Data **8**, 91-92. (10.1016/j.gdata.2016.04.002) 27222807 PMC4856825

[49] Naumenko SA et al. 2017 Transcriptome-based phylogeny of endemic Lake Baikal amphipod species flock: fast speciation accompanied by frequent episodes of positive selection. Mol. Ecol. **26**, 536-553. (10.1111/mec.13927) 27859915

[50] Cogne Y et al. 2019 *De novo* transcriptomes of 14 gammarid individuals for proteogenomic analysis of seven taxonomic groups. Sci. Data **6**, 184. (10.1038/s41597-019-0192-5) 31562330 PMC6764967

[51] Caputo DR, Robson SC, Werner I, Ford AT. 2020 Complete transcriptome assembly and annotation of a critically important amphipod species in freshwater ecotoxicological risk assessment: *Gammarus fossarum*. Environ Int. **137**, 105319. (10.1016/j.envint.2019.105319) 32028177

[52] Collins M, Clark MS, Spicer JI, Truebano M. 2021 Transcriptional frontloading contributes to cross-tolerance between stressors. Evol. Appl. **14**, 577-587. (10.1111/eva.13142) 33664796 PMC7896706

[53] Lipaeva P et al. 2021 Different ways to play it cool: transcriptomic analysis sheds light on different activity patterns of three amphipod species under long-term cold exposure. Mol. Ecol. **30**, 5735-5751. (10.1111/mec.16164) 34480774

[54] Neuparth T et al. 2020 Transcriptomic data on the transgenerational exposure of the keystone amphipod *Gammarus locusta* to simvastatin. Data Br. **32**, 106248. (10.1016/j.dib.2020.106248) PMC7481811 32944603

[55] Drozdova P et al. 2019 Comparison between transcriptomic responses to short-term stress exposures of a common Holarctic and endemic Lake Baikal amphipods. BMC Genom. **20**, 712. (10.1186/s12864-019-6024-3) PMC6743106 31519144

[56] Hashimoto K, Yoshizawa AC, Okuda S, Kuma K, Goto S, Kanehisa M. 2008 The repertoire of desaturases and elongases reveals fatty acid variations in 56 eukaryotic genomes. J. Lipid Res. **49**, 183-191. (10.1194/jlr.M700377-JLR200) 17921532

[57] Eyres I, Boschetti C, Crisp A, Smith TP, Fontaneto D, Tunnacliffe A, Barraclough TG. 2015 Horizontal gene transfer in bdelloid rotifers is ancient, ongoing and more frequent in species from desiccating habitats. BMC Biol. **13**, 90. (10.1186/s12915-015-0202-9) 26537913 PMC4632278

[58] Katoh K, Rozewicki J, Yamada KD. 2019 MAFFT online service: multiple sequence alignment, interactive sequence choice and visualization. Brief. Bioinform. **20**, 1160-1166. (10.1093/bib/bbx108) 28968734 PMC6781576

[59] Capella-Gutiérrez S, Silla-Martínez JM, Gabaldón T. 2009 trimAl: a tool for automated alignment trimming in large-scale phylogenetic analyses. Bioinformatics **25**, 1972-1973. (10.1093/bioinformatics/btp348) 19505945 PMC2712344

[60] Kozlov AM, Darriba D, Flouri T, Morel B, Stamatakis A. 2019 RAxML-NG: a fast, scalable and user-friendly tool for maximum likelihood phylogenetic inference. Bioinformatics **35**, 4453-4455. (10.1093/bioinformatics/btz305) 31070718 PMC6821337

[61] Flouri T, Izquierdo-Carrasco F, Darriba D, Aberer AJ, Nguyen LT, Minh BQ, Von Haeseler A, Stamatakis A. 2015 The phylogenetic likelihood library. Syst. Biol. **64**, 356-362. (10.1093/sysbio/syu084) 25358969 PMC4380035

[62] Darriba D, Posada D, Kozlov AM, Stamatakis A, Morel B, Flouri T. 2020 ModelTest-NG: a new and scalable tool for the selection of DNA and protein evolutionary models. Mol. Biol. Evol. **37**, 291-294. (10.1093/molbev/msz189) 31432070 PMC6984357

[63] Bojko J, Bącela-Spychalska K, Stebbing PD, Dunn AM, Grabowski M, Rachalewski M, Stentiford GD. 2017 Parasites, pathogens and commensals in the ‘low-impact’ non-native amphipod host *Gammarus roeselii*. Parasit. Vectors **10**, 193. (10.1186/s13071-017-2108-6) 28427445 PMC5397875

[64] De Smet WH, Verolet M. 2016 Epibiotic rotifers of *Gammarus pulex* (L.) (Crustacea, Amphipoda), with descriptions of two new species and notes on the terminology of the trophi. Zootaxa **4107**, 301-320. (10.11646/zootaxa.4107.3.1) 27394822

[65] Bojko J, Ovcharenko M. 2019 Pathogens and other symbionts of the Amphipoda: taxonomic diversity and pathological significance. Dis. Aquat. Org. **136**, 3-36. (10.3354/dao03321) 31575832

[66] Bushmanova E, Antipov D, Lapidus A, Prjibelski AD. 2019 rnaSPAdes: a *de novo* transcriptome assembler and its application to RNA-Seq data. GigaScience **8**, giz100. (10.1093/gigascience/giz100) 31494669 PMC6736328

[67] Camacho C, Coulouris G, Avagyan V, Ma N, Papadopoulos J, Bealer K, Madden TL. 2009 BLAST+: architecture and applications. BMC Bioinform. **10**, 421. (10.1186/1471-2105-10-421) PMC2803857 20003500

[68] Hebert PDN, Cywinska A, Ball SL, De Waard JR. 2003 Biological identifications through DNA barcodes. Proc. R. Soc. Lond. B **270**, 313-321. (10.1098/rspb.2002.2218) PMC1691236 12614582

[69] Kabeya N, Gür I, Oboh A, Evjemo JO, Malzahn AM, Hontoria F, Navarro JC, Monroig Ó. 2020 Unique fatty acid desaturase capacities uncovered in *Hediste diversicolor* illustrate the roles of aquatic invertebrates in trophic upgrading. Phil. Trans. R. Soc. B **375**, 20190654. (10.1098/rstb.2019.0654) 32536307 PMC7333967

[70] Monroig Ó, Tocher DR, Hontoria F, Navarro JC. 2013 Functional characterisation of a Fads2 fatty acyl desaturase with Δ6/Δ8 activity and an Elovl5 with C16, C18 and C20 elongase activity in the anadromous teleost meagre (*Argyrosomus regius*). Aquaculture **412**, 14-22. (10.1016/j.aquaculture.2013.06.032)

[71] Folch J, Lees M, Sloane Stanley GH. 1957 A simple method for the isolation and purification of total lipides from animal tissues. J. Biol. Chem. **226**, 497-509. (10.1016/S0021-9258(18)64849-5) 13428781

[72] Ribes-Navarro A, Alberts-Hubatsch H, Monroig Ó, Hontori F, Navarro JC. 2022 Effects of diet and temperature on the fatty acid composition of the gammarid *Gammarus locusta* fed alternative terrestrial feeds. Front. Mar. Sci. **9**, 931991. (10.3389/fmars.2022.931991)

[73] Surm JM, Prentis PJ, Pavasovic A. 2015 Comparative analysis and distribution of omega-3 LC-PUFA biosynthesis genes in marine molluscs. PLoS ONE **10**, e0136301. (10.1371/journal.pone.0136301) 26308548 PMC4550275

[74] Surm JM, Toledo TM, Prentis PJ, Pavasovic A. 2018 Insights into the phylogenetic and molecular evolutionary histories of *Fad* and *Elovl* gene families in Actiniaria. Ecol. Evol. **8**, 5323-5335. (10.1002/ece3.4044) 29938056 PMC6010785

[75] Van Der Burgt A, Severing E, De Wit PJ, Collemare J. 2012 Birth of new spliceosomal introns in fungi by multiplication of introner-like elements. Curr. Biol. **22**, 1260-1265. (10.1016/j.cub.2012.05.011) 22658596

[76] Ishikawa A et al. 2019 A key metabolic gene for recurrent freshwater colonization and radiation in fishes. Science **364**, 886-889. (10.1126/science.aau5656) 31147520

[77] Matsushita Y et al. 2020 Flatfishes colonised freshwater environments by acquisition of various DHA biosynthetic pathways. Commun. Biol. **3**, 516. (10.1038/s42003-020-01242-3) 32948803 PMC7501227

[78] Ricci C, Melone G. 2000 Key to the identification of the genera of bdelloid rotifers. Hydrobiologia **418**, 73-80. (10.1023/A:1003840216827)

[79] Davidson NM, Oshlack A. 2014 Corset: enabling differential gene expression analysis for *de novo* assembled transcriptomes. Genome Biol. **15**, 410. (10.1186/s13059-014-0410-6) 25063469 PMC4165373

[80] Haas BJ et al. 2013 *De novo* transcript sequence reconstruction from RNA-seq using the Trinity platform for reference generation and analysis. Nat. Protoc. **8**, 1494-1512. (10.1038/nprot.2013.084) 23845962 PMC3875132

[81] Grabherr MG et al. 2011 Full-length transcriptome assembly from RNA-Seq data without a reference genome. Nat. Biotechnol. **29**, 644-652. (10.1038/nbt.1883) 21572440 PMC3571712

[82] Nowell RW, Wilson CG, Almeida P, Schiffer PH, Fontaneto D, Becks L, Rodriguez F, Arkhipova IR, Barraclough TG. 2021 Evolutionary dynamics of transposable elements in bdelloid rotifers. Elife **10**, e63194. (10.7554/eLife.63194) 33543711 PMC7943196

[83] Debortoli N, Li X, Eyres I, Fontaneto D, Hespeels B, Tang CQ, Flot JF, Van Doninck K. 2016 Genetic exchange among bdelloid rotifers is more likely due to horizontal gene transfer than to meiotic sex. Curr. Biol. **26**, 723-732. (10.1016/j.cub.2016.01.031) 26948882

[84] Hespeels B, Li X, Flot J-F, Pigneur L-M, Malaisse J, Da Silva C, Van Doninck K. 2015 Against all odds: trehalose-6-phosphate synthase and trehalase genes in the bdelloid rotifer *Adineta vaga* were acquired by horizontal gene transfer and are upregulated during desiccation. PLoS ONE **10**, e0131313. (10.1371/journal.pone.0131313) 26161530 PMC4498783

[85] Gladyshev EA, Meselson M, Arkhipova IR. 2008 Massive horizontal gene transfer in bdelloid rotifers. Science **320**, 1210-1213. (10.1126/science.1156407) 18511688

[86] Flot J-F et al. 2013 Genomic evidence for ameiotic evolution in the bdelloid rotifer *Adineta vaga*. Nature **500**, 453-457. (10.1038/nature12326) 23873043

[87] Boschetti C, Carr A, Crisp A, Eyres I, Wang-Koh Y, Lubzens E, Barraclough TG, Micklem G, Tunnacliffe A. 2012 Biochemical diversification through foreign gene expression in bdelloid rotifers. PLoS Genet. **8**, e1003035. (10.1371/journal.pgen.1003035) 23166508 PMC3499245

[88] Villa TG, Viñas M. 2019 Horizontal gene transfer. Berlin, Germany: Springer International Publishing.

[89] Pontarotti P. 2014 Evolutionary biology: genome evolution, speciation, coevolution and origin of life. Berlin, Germany: Springer International Publishing.

[90] Syvanen M. 1994 Horizontal gene transfer: evidence and possible consequences. Annu. Rev. Genet. **28**, 237-261. (10.1146/annurev.ge.28.120194.001321) 7893125

[91] Arndt H. 1993 Rotifers as predators on components of the microbial web (bacteria, heterotrophic flagellates, ciliates)—a review. Hydrobiologia **255**, 231-246. (10.1007/BF00025844)

[92] Von Der Heyden S, Cavalier-Smith T. 2005 Culturing and environmental DNA sequencing uncover hidden kinetoplastid biodiversity and a major clade within ancestrally freshwater *Neobodo designis*. Int. J. Syst. Evol. Microbiol. **55**, 2605-2621. (10.1099/ijs.0.63606-0) 16280534

[93] Mukherjee I, Hodoki Y, Okazaki Y, Fujinaga S, Ohbayashi K, Nakano SI. 2019 Widespread dominance of kinetoplastids and unexpected presence of diplonemids in deep freshwater lakes. Front. Microbiol. **10**, 2375. (10.3389/fmicb.2019.02375) 31681232 PMC6805782

[94] José De Paggi SB, Marinone MC, Küppers GC, Claps MC, Paggi JC. 2022 Taxonomic diversity of the freshwater zooplankton in Argentina: a review. Limnologica **100**, 126029. (10.1016/j.limno.2022.126029)

[95] Schwartz SH, Silva J, Burstein D, Pupko T, Eyras E, Ast G. 2008 Large-scale comparative analysis of splicing signals and their corresponding splicing factors in eukaryotes. Genome Res. **18**, 88-103. (10.1101/gr.6818908) 18032728 PMC2134773

[96] Worden AZ et al. 2009 Green evolution and dynamic adaptations revealed by genomes of the marine picoeukaryotes *Micromonas*. Science **324**, 268-272. (10.1126/science.1167222) 19359590

[97] Gozashti L, Roy SW, Thornlow B, Kramer A, Ares M, Corbett-Detig R. 2022 Transposable elements drive intron gain in diverse eukaryotes. Proc. Natl Acad. Sci. USA **119**, e2209766119. (10.1073/pnas.2209766119) 36417430 PMC9860276

[98] Denoeud F et al. 2010 Plasticity of animal genome architecture unmasked by rapid evolution of a pelagic tunicate. Science **330**, 1381-1385. (10.1126/science.1194167) 21097902 PMC3760481

[99] Torriani SFF, Stukenbrock EH, Brunner PC, Mcdonald BA, Croll D. 2011 Evidence for extensive recent intron transposition in closely related fungi. Curr. Biol. **21**, 2017-2022. (10.1016/j.cub.2011.10.041) 22100062

[100] Bouck GB. 1993 The biology of free-living heterotrophic flagellates. J. Eukaryot. Microbiol. **40**, 113-114. (10.1111/j.1550-7408.1993.tb04890.x)

[101] Arndt H, Dietrich D, Auer B, Cleven E, Gräfenhan T, Weitere M, Mylnikov A. 2000 Functional diversity of heterotrophic flagellates in aquatic ecosystems. In The flagellates (eds BSC Leadbeater, JC Green), pp. 240-268. London, UK: Taylor & Francis.

[102] Tripodi KEJ, Buttigliero LV, Altabe SG, Uttaro AD. 2006 Functional characterization of front-end desaturases from trypanosomatids depicts the first polyunsaturated fatty acid biosynthetic pathway from a parasitic protozoan. FEBS J. **273**, 271-280. (10.1111/j.1742-4658.2005.05049.x) 16403015

[103] Park WJ, Kothapalli KSD, Lawrence P, Tyburczy C, Brenna JT. 2009 An alternate pathway to long-chain polyunsaturates: the *FADS2* gene product Δ8-desaturates 20:2*n-*6 and 20:3*n-*3. J. Lipid Res. **50**, 1195-1202. (10.1194/jlr.M800630-JLR200) 19202133 PMC2681401

[104] Monroig Ó, Li Y, Tocher DR. 2011 Delta-8 desaturation activity varies among fatty acyl desaturases of teleost fish: high activity in δ-6 desaturases of marine species. Comp. Biochem. Physiol. B Biochem. Mol. Biol. **159**, 206-213. (10.1016/j.cbpb.2011.04.007) 21571087

[105] Ran Z, Xu J, Liao K, Li S, Chen S, Yan X. 2018 Biosynthesis of polyunsaturated fatty acids in the razor clam *Sinonovacula constricta*: characterization of Δ5 and Δ6 fatty acid desaturases. J. Agric. Food Chem. **66**, 4592-4601. (10.1021/acs.jafc.8b00968) 29676149

[106] Wagner A. 1998 The fate of duplicated genes: loss or new function? BioEssays **20**, 785-788. (10.1002/(sici)1521-1878(199810)20:10<785::aid-bies2>3.0.co;2-m) 10200118

[107] Birchler JA, Yang H. 2022 The multiple fates of gene duplications: deletion, hypofunctionalization, subfunctionalization, neofunctionalization, dosage balance constraints, and neutral variation. Plant Cell **34**, 2466-2474. (10.1093/plcell/koac076) 35253876 PMC9252495

[108] Vogan AA, Higgs PG. 2011 The advantages and disadvantages of horizontal gene transfer and the emergence of the first species. Biol. Direct **6**, 1. (10.1186/1745-6150-6-1) 21199581 PMC3043529

[109] Husnik F, Mccutcheon J. 2018 Functional horizontal gene transfer from bacteria to eukaryotes. Nat. Rev. Microbiol **16**, 67-79. (10.1038/nrmicro.2017.137) 29176581

[110] Colombo SM, Wacker A, Parrish CC, Kainz MJ, Arts MT. 2016 A fundamental dichotomy in long-chain polyunsaturated fatty acid abundance between and within marine and terrestrial ecosystems. Environ. Rev. **25**, 163-174. (10.1139/er-2016-0062)

[111] Ribes-Navarro A, Kabeya N, Castro LFC, Gomes-dos-Santos A, Fonseca MM, Alberts-Hubatsch H, Hontoria F, Navarro JC, Monroig Ó. 2023 Examination of gammarid transcriptomes reveals a widespread occurrence of key metabolic genes from epibiont bdelloid rotifers in freshwater species. Figshare. (10.6084/m9.figshare.c.6858619) PMC10597677 37875161

